# Bilateral Transcranial Magnetic Stimulation on DLPFC Changes Resting State Networks and Cognitive Function in Patients With Bipolar Depression

**DOI:** 10.3389/fnhum.2018.00356

**Published:** 2018-09-05

**Authors:** Reza Kazemi, Reza Rostami, Sanaz Khomami, Golnaz Baghdadi, Mehdi Rezaei, Masahiro Hata, Yasunori Aoki, Ryouhei Ishii, Masao Iwase, Paul B. Fitzgerald

**Affiliations:** ^1^Cognitive Lab, Department of Psychology, University of Tehran, Tehran, Iran; ^2^Atieh Clinical Neuroscience Center, Tehran, Iran; ^3^Department of Psychology, University of Tehran, Tehran, Iran; ^4^Department of Biomedical Engineering, Amirkabir University of Technology, Tehran, Iran; ^5^Behavioral Sciences Research Center, Life Style Institute, Baqiyatallah University of Medical Sciences, Tehran, Iran; ^6^Department of Psychiatry, Graduate School of Medicine, Osaka University, Osaka, Japan; ^7^Epworth Healthcare, Epworth Clinic Camberwell, Victoria Australia and Monash Alfred Psychiatry Research Centre, Central Clinical School, Monash University, Melbourne, VIC, Australia

**Keywords:** sensorimotor network, default mode network, bipolar depression, bilateral transcranial magnetic stimulation, low-resolution electromagnetic tomography, resting state networks, cognitive functions

## Abstract

**Introduction:** Bipolar patients have abnormalities in cognitive functions and emotional processing. Two resting state networks (RSNs), the default mode network (DMN) and the sensorimotor network (SMN), play a decisive role in these two functions. Dorsolateral prefrontal cortex (DLPFC) is one of the main areas in the central executive network (CEN), which is linked to the activities of each of the two networks. Studies have found DLPFC abnormalities in both hemispheres of patients with bipolar depression. We hypothesized that the bilateral repetitive transcranial magnetic stimulation (rTMS) of DLPFC would produce changes in the activity of both the SMN and DMN as well as relevant cognitive function in patients with bipolar depression that responded to treatment.

**Methods:** 20 patients with bipolar depression underwent 10 sessions of 1 Hz rTMS on right DLPFC with subsequent 10 Hz rTMS on left DLPFC. Changes in electroencephalography resting networks between pre and post rTMS were evaluated utilizing low-resolution electromagnetic tomography (eLORETA). Depression symptom was assessed using the Beck Depression Inventory (BDI-II) and cognitive function was assessed by Verbal Fluency Test (VFT), Rey Auditory Verbal Learning Test (RAVLT), Stroop Test, and Wisconsin Card Sorting Test (WCST).

**Results:** Responders to rTMS showed significantly lower DMN activity at baseline and a significant decrease in SMN connectivity after treatment. Non-responders did not significantly differ from the control group at the baseline and they showed higher activity in the SMN, visual network, and visual perception network compared to control group following treatment. Bilateral rTMS resulted in significant changes in the executive functions, verbal memory, and depression symptoms. No significant changes were observed in selective attention and verbal fluency.

**Conclusion:** Bilateral stimulation of DLPFC, as the main node of CEN, results in changes in the activity of the SMN and consequently improves verbal memory and executive functions in patients with bipolar depression.

## Introduction

Emotional processing (Wessa and Linke, 2009) and cognitive functions (Torrent et al., 2006) are two areas known to be impaired in individuals with bipolar disorder. Those with bipolar disorder may be unable to use cognitive functions to regulate and maintain emotional states. This can lead to a dysfunction in their emotional processing and emotion regulation (Keener and Phillips, 2007; Phillips and Vieta, 2007). Studies on resting state neural networks in bipolar disorder have identified abnormalities in four networks, including the default mode network (DMN; Fan et al., 2012), central executive network (CEN; Baker et al., 2014), salience network (SN; Lopez-Larson et al., 2017), and sensorimotor network (SMN; Martino et al., 2016). The DMN and SMN have a major role in emotional and cognitive processing. Several studies have focused on DMN abnormalities in individuals with bipolar disorder (Calhoun et al., 2008; Allin et al., 2010; Öngür et al., 2010). It has been reported that DMN deactivation plays an important role in cognitive functions (Daselaar et al., 2004; Shulman et al., 2007); decreased DMN activity is correlated with successful functioning in various cognitive domains (Anticevic et al., 2012). One trial identified greater activity in the areas of middle temporal gyrus, middle frontal gyrus, and caudate in individuals with bipolar disorder compared to healthy controls (Fan et al., 2012). Similarly, reduced DMN deactivation has been observed in patients with bipolar depression during cognitive tasks (Fernández-Corcuera et al., 2013).

Research has also suggested the CEN and DMN act conversely (26), with the SN mediating activity between the two (Goulden et al., 2014). Both CEN and SN negatively regulate DMN function (Sridharan et al., 2008; Murphy et al., 2009). A recent study has shown that inhibition of a major node in CEN by 1 Hz repetitive transcranial magnetic stimulation (rTMS) leads to disinhibition of DMN. Conversely, stimulation by single pulse TMS leads to a negative connectivity of DMN with CEN and SN (Chen et al., 2013). SMN has an important role in emotional functions [e.g., emotion discrimination (Banissy et al., 2010) and emotion recognition (Wood et al., 2016; Davis et al., 2017)] and cognitive functions [e.g., working memory (D’Esposito and Postle, 2015) and social cognition (Pineda, 2008)]. Recently, it has been argued that abnormality in interhemispheric activities in SMN is the basis of emotion processing dysfunction in patients with bipolar disorder (Ishida et al., 2017). In fact, in a number of mental health disorders, the interaction between SMN and DMN has been shown to be dysfunctional (Chenji et al., 2016).

Dorsolateral prefrontal cortex (DLPFC) is a major node in the CEN, associated with both cognitive (Townsend et al., 2010) and emotional (Hassel et al., 2008) abnormalities in bipolar disorder. Abnormality of DLPFC function has been identified in both hemispheres in patients with bipolar depression (Brooks et al., 2009). Decreased DLPFC metabolism has been reported in some studies, (Baxter et al., 1989; Martinot et al., 1990) while metabolic increase is reported in others (Ketter et al., 2001). rTMS to the DLPFC is expected to have an impact on both the SMN and the DMN networks, particularly to the pre-SMA (Wang et al., 2005; Nachev et al., 2008) and mPFC (Chai et al., 2011), respectively. As discussed, both of these interconnected networks are involved in bipolar disorder, with reduced SMN activity and increased DMN activity evident in bipolar depression (Martino et al., 2016).

A recently published meta-analysis suggested rTMS is a safe and relatively effective therapy to treat bipolar depression (McGirr et al., 2016). There is a very low risk for treatment-emergent affective switches, while no increased risk of future manic episodes from a course of active rTMS treatment has been observed (McGirr et al., 2016). Given the involvement of the DMN, SMN, and DLPFC in bipolar depression (Fernández-Corcuera et al., 2013) and that DLPFC stimulation can affect the DMN and SMN, the current study proposed that rTMS provided to the DLPFC would alter DMN and SMN function, with subsequent improvement in cognitive function and emotional processing relevant to bipolar depression. Given the reported involvement of the DLPFC in both hemispheres in patients with bipolar depression (Brooks et al., 2009), sequential bilateral rTMS was selected as the intervention for this study.

Electroencephalography (EEG) was selected as a tool to obtain new insights on neurophysiological features in patients with bipolar depression, especially in regards to the role of the DMN and SMN in mediating clinical response. Changes in EEG resting state networks (RSNs) between pre and post rTMS were explored utilizing EEG functional network analysis, evaluated by exact low-resolution electromagnetic tomography (eLORETA; Pascual-Marqui et al., 2011). eLORETA is a three-dimensional, discrete, linear, and weighted minimal norm inverse solution method. It is uniquely endowed with the property of exact localization to a test point source at any location, albeit with low spatial resolution. Because of the principles of linearity and superposition, the method produces a low-resolution estimate of any distribution of electric neuronal activity. In a detailed and exhaustive comparison with other competing linear inverse solution methods, it was shown that eLORETA has improved localization properties in the presence of noise and in multiple source situations (Pascual-Marqui et al., 2011). In a previous study utilizing resting state EEG data of 80 healthy subjects, five resting state independent networks were identified with the eLORETA system (Aoki et al., 2015).

Hypotheses of the current study were therefore (1) patients with bipolar depression will show abnormal DMN connectivity compared to controls; (2) DLPFC rTMS will produce changes in regions of the DMN and SMN which contribute to improvement of cognitive functions and clinical symptoms in responders to rTMS; and (3) DLPFC rTMS will produce changes in DMN and SMN connectivity in patients with bipolar depression who respond to treatment.

## Materials and Methods

This was an open-label study in which 20 patients with bipolar disorder received 10 sessions of sequential bilateral rTMS, one session a day, 6 days a week. Patients were evaluated at baseline (pre-treatment) and at the end of the treatment course (post-treatment). Resting EEG data from these 20 patients was compared to data from 80 healthy controls collected in a previous study (38).

### Participants

The clinical sample consisted of twenty patients (8 men and 12 women; M ± SD = 28.65, age range of 16–47) referred to the Atieh Clinical Neuroscience Center (Tehran, Iran) from April to September 2015. All patients had a diagnosis of bipolar disorder, and were experiencing a current depressive episode as verified by a psychiatrist based on DSM-IV-TR criteria. The inclusion criteria were (1) age range of 16–70 years, (2) diagnosis of bipolar depression confirmed by a psychiatrist and based on DSM-IV-TR, (3) current treatment under supervision of a psychiatrist, (4) a score higher than 14 (mild depression) on the Beck Depression Inventory (BDI-II; Beck et al., 1996), and (5) unchanged medication regime during the treatment process. The study exclusion criteria were (1) a history of rTMS treatment for any disorder, (2) presence of intracranial implants (such as shunts, irritations, electrodes) or any other metal object inside or near the head (e.g. mouth) which could not be removed, (3) cardiac pacemaker, (4) acute heart disease, (5) a history of epilepsy or seizure in the individual or first degree relatives, (6) a history of head trauma, and (7) pregnant or breastfeeding women. Medication was unchanged from a month before starting the treatment until the end of the course of rTMS. If the treating psychiatrist identified any need to change a patient’s medication, the patient was excluded from the study.

Resting state EEG data, collected from 80 healthy control participants in a previous study (27), were utilized for comparison purposes. The control sample consisted of 57 males and 23 females (mean age = 44, standard deviation = 20) without any history of neurological or psychiatric disorder. Control participants 60 years of age or older were screened for global cognitive deficits [i.e., mini-mental state examination (MMSE) and clinical dementia rating (CDR)]. A CDR score of zero was obtained for all screened participants and a median MMSE score of 30 (interquartile range; 29–30). A 120-s window of recorded EEG was selected and artifact rejected after strict visual inspections of the certified electroencephalographers.

**Table 1** shows demographic and clinical information for clinical and control participants. Informed consent was obtained prior to commencement of this trial from all participants who received TMS. The study was approved by the University of Tehran ethics committee.

**Table 1:** Demographic and clinical characteristics of study participants and paired t-test results in test variables of Wisconsin Card Sorting Test, Rey Auditory Verbal Learning Test, Verbal Fluency Test, Stroop Test, and Beck Depression Inventory.

	*N* rTMS group		*N* control group					
**Sex**								
Male	8 (40%)		57 (71/25%)					
Female	12 (60%)		23 (28/75%)					
**Medication**								
Tricyclics	11 (24/4%)							
Selective serotonin reuptake inhibitor	4 (9%)							
Atypical antipsychotics	14 (31/1%)							
Mood stabilizers	16 (35/5%)							

	**Mean**	**SD**	**Mean**	**SD**	***t***	***df***	***P***	**Eta squared**

**Executive function**								
Preservative errors (WCST)	4.95	2.76	2.25	2.40	4.64	19	0.0001	0.51
Total errors (WCST)	14.45	4.80	11.80	4.84	2.38	19	0.03	0.23
Total efforts (WCST)	57.35	4.20	53.55	5.50	2.52	19	0.02	0.25
Completed number of categories (WCST)	4.70	1.71	5.10	1.48	-1.32	19	0.20	0.08
Phonetic fluency	34.65	15.45	37.40	14.73	-1.28	19	0.21	0.07
Semantic fluency	53.95	9.70	57.05	12.37	-1.72	19	0.10	0.14
**Memory and verbal learning**								
Total recall	12.45	1.82	13.95	1.57	-4.56	19	0.0001	0.52
Immediate recall	10.55	2.80	13.45	1.60	-5.66	19	0.0001	0.62
Delayed recall	10.88	3.02	13.30	2.57	-3.84	19	0.001	0.43
Recognition	13.90	1.21	14.45	0.88	-1.99	19	0.06	0.17
**Attention and focus**								
Stroop interference	0.77	2.10	0.50	1.85	0.63	19	0.54	0.002
**Depression symptoms**								
Beck depression inventory	30.15	10.05	15.25	8.37	4.77	19	0.0001	0.54

### rTMS

Repetitive transcranial magnetic stimulation was administered with a Magstim Rapid 2 machine (Magstim Company Ltd., Whitland, United Kingdom) and a 70-mm figure-of-eight coil (air film coil). rTMS treatment was applied on F3 and F4 EEG regions based on the international 10-20 system. Right DLPFC stimulation was applied at 1 Hz for a 10-s train of stimulation, 2-s inter-train interval, and a total of 150 pulse trains. This resulted in 1500 pulses per session for a total of 15,000 pulses to the right DLPFC over 10 sessions. Within each session, right side stimulation was immediately followed by left DLPFC stimulation at a frequency of 10 Hz, 5 s of stimulation, 10-second inter-train interval, and 75 pulse trains. This resulted in 3750 pulses per session and a total of 37,500 pulses over 10 sessions to left DLPFC.

Resting motor threshold (RMT) was determined prior to treatment. RMT is defined as the minimum intensity required to stimulate the motor cortex and lead to a contraction in the abductor policies brevis (APB) muscle. Stimulation at threshold should cause APB muscle contraction in at least five out of 10 attempts. Treatment stimulation intensity was set at 120% of the RMT on the right side and 100% of the RMT on the left side.

### EEG Recording

Electroencephalography data were recorded by a 19-channel amplifier (Mitsar, Russia) using an ElectroCap (ElectroCap, Inc, OH). Electrodes were located on a cap based on a 10-20 system. A1+A2 electrode was used as the reference. Electrode impedance was kept below 5 kΩ and the sampling rate was 250 Hz. EEG was recorded for 5 min while patients were resting in an acoustics room with closed eyes. EEG data were filtered using a band-pass filter (0.3–40 Hz). Electroocular artifacts were removed by setting the amplitude threshold to ±70 μV. Independent component analysis (ICA) was also performed to remove muscle artifacts. After artifact removal an interval of 60 s of the data for each subject were used for further analyses.

### eLORETA Network Analysis

The eLORETA brain model and electrode coordinate system are based on the Montreal Neurological Institute average MRI brain map (MNI 152; Mazziotta et al., 2001). The solution space is limited to the cortical gray matter, comprising 6239 voxels of 5-mm^3^ space resolution. The validity of eLORETA tomography as a reliable and effective tool for exploring brain activities has been confirmed by several studies using intracranial EEG (Zumsteg et al., 2006), PET (Dierks et al., 2000), structural MRI (Worrell et al., 2000), and fMRI (Vitacco et al., 2002; Mulert et al., 2004). eLORETA images in the current study were evaluated in the following five frequency bands: delta (2–4 Hz), theta (4–8 Hz), alpha (8–13 Hz), beta (13–30 Hz), and gamma (30–60 Hz).

**FIGURE 1:**
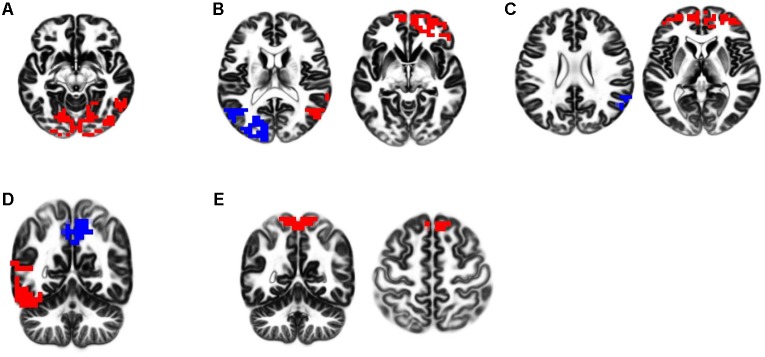
eLORETA-neurophysiological independent components (ICs). Five neurophysiological network activities were identified in the previous study. In respective figures, red and blue voxels indicate increasing and decreasing in power, respectively, with increasing ICs activities. The definitions of these networks are described as follows. **(A)** IC-4; the visual network in alpha frequency band. **(B)** IC-5; dual-process of visual perception network, characterized by a negative correlation between the right ventral visual pathway (VVP) in alpha and beta frequency bands and left posterior dorsal visual pathway (DVP) in alpha frequency band. **(C)** IC-6; self-referential processing network (DMN), characterized by a negative correlation between the medial prefrontal cortex (mPFC) in beta frequency band and right temporoparietal junction (TPJ) in alpha frequency band. **(D)** IC-9; dual-process of memory perception network, functionally related to a negative correlation between the left VVP and the precuneus in alpha frequency band. **(E)** IC-10; sensorimotor network (SMN) in beta and gamma frequency bands.

The 60 s of artifact-free EEG from all participants’ recordings were fragmented into 2-s fragments offline. The processed 2-s artifact-free EEG fragments were analyzed with eLORETA software, exploring functional EEG activity based on the five RSNs reported in the previous study (Aoki et al., 2015; see **Figure 1**). These networks included (1) independent component 4 (IC-4): the visual network in alpha frequency band; (2) IC-5: dual-process visual perception network, characterized by a negative correlation between the right ventral visual pathway (VVP) in alpha and beta frequency bands and left posterior dorsal visual pathway (DVP) in alpha frequency band; (3) IC-6: self-referential processing network (DMN), characterized by a negative correlation between the medial prefrontal cortex (mPFC) in beta frequency band and right temporoparietal junction (TPJ) in alpha frequency band; (4) IC-9: dual-process of memory perception network, functionally related to a negative correlation between the left VVP and the precuneus in alpha frequency band; and (5) IC-10: SMN in beta and gamma frequency bands.

Detailed explanations of the methodology utilized in this RSN analysis can be found in the original Aoki et al. (2015) study described above. However, a brief description of eLORETA-ICA, by which these five RSNs were obtained is provided here. ICA is a mathematical and statistical technique which disintegrates mixed signals into statistically independent components. Electrical activities of desired vertices (cortical solutions) are computed by eLORETA using scalp EEG recordings. Cortical solutions for subjects over frequency bands are arranged in data matrices with the format: subject × frequency band × cortical solutions. Data matrices are consequently processed by a group ICA application embedded in eLORETA (Pascual-Marqui and Biscay-Lirio, 2011), yielding a set of independent components. The independence of these components was maximized based on fourth-order cumulant as its difference criterion (Cardoso, 1989; Cichocki and Amari, 2002). Subsequently, independent components with red and blue colored brain maps (red and blue indicate increasing and decreasing cortical activities respectively, as independent component activities are increased) were derived in total power order. Therefore, differences in functional network construction in two sets of EEG data can be evaluated by comparing their corresponding independent components’ coefficients.

Thus, in this study, we used these five networks to investigate the difference of functional network constituents between patients with bipolar depression and healthy controls (Aoki et al., 2015). To explore difference in network coefficients we calculated z-scores. All coefficients were adjusted by age in linear regression based on Aoki et al. (2015). The significance level was set at *p* = 0.05 following Bonferroni correction.

### Cognitive and Clinical Assessment

The primary outcome measure was cognitive function: executive functioning, selective attention, and verbal memory was assessed before and after treatment by the (1) Verbal Fluency Test (VFT) (Lezak, 2004), (2) Rey Auditory Verbal Learning Test (RAVLT) (Jafari et al., 2010), (3) Stroop Test (Stroop, 1935), and (4) Wisconsin Card Sorting Test (WCST; Nelson, 1976). The secondary outcome measure was response rate (50% or greater reduction in mean BDI-II scores from baseline to end of treatment). Paired *t*-tests were used to evaluate change in cognitive functions and depressive symptoms.

## Results

All participants with bipolar disorder completed a course of rTMS treatment. No side effects of rTMS were observed. Two patients were not able to participate in post-treatment EEG recording because of issues undertaking the EEG testing.

### Effect of rTMS on Cognitive Functions and Depressive Symptoms

#### Effects of rTMS on Cognitive Functions

*T*-tests (**Table 1**) showed a decrease in preservation errors [*t*_(19)_ = 4.64, *P* < 0.0001], total errors on the WCST [*t*_(19)_= 2.38, *P* = 0.03], and total efforts [*t*_(19)_= 2.52, *P* = 0.02] following a course of rTMS. However, there was no significant increase in the completed number of WCST categories, or any change in verbal fluency, or change on the STROOP test (all *p* > 0.05). Verbal memory was enhanced post-rTMS, evident in total recall (*P* = 0.0001), immediate recall (*P* = 0.0001), and delayed recall (*p* = 0.0001). No significant effect on recognition memory was seen (*p* > 0.05).

#### Effects of rTMS on Depressive Symptoms

A decrease in depression symptoms was evident from baseline to treatment end [*t*_(19)_ = 4.77, *P* < 0.0001). Eleven of 20 patients (55%) met response criteria (≥50% reduction in BDI-II score), while three of 20 patients met remission criteria (final BDI-II score < 8).

### eLORETA Network Analysis

#### Baseline Comparison of Patients vs Healthy Controls

At baseline, the only difference between the patients (*n* = 18) and the control group was in the DMN (mPFC and TPJ). The patients with BPAD at baseline exhibited significantly less activity in IC-6 coefficient (self-referential processing network, characterized by a negative correlation between the mPFC in beta frequency band and right TPJ in alpha frequency band) compared to the controls (*p* = 0.007).

#### Responders and Non-responders vs Controls Before Treatment

Similar to the overall sample, the only difference between responders (*n* = 10) and the control group prior to treatment was in DMN (mPFC and TPJ; *p* = 0.0009). Non-responders (*n* = 8) did not differ from the control group across any networks. Compared with healthy controls prior to treatment, responders exhibited a significant lack of activity in IC-6 coefficient (self-referential processing network, characterized by a negative correlation between the mPFC in beta frequency band and right TPJ in alpha frequency band). Non-responders (*n* = 8) showed no significant differences from controls prior to treatment.

#### Responders Post-treatment

After treatment, the only difference between responders and the control group was in the SMN. Post-treatment, responders exhibited significantly less activity in the IC-10 coefficient (SMN in beta and gamma frequency bands) compared with the model of healthy controls (*p* = 0.012).

#### Non-responders Post-treatment

Non-responders exhibited significantly higher activity in IC-4 coefficient (the visual network in alpha frequency band; *p* = 0.015) and IC-10 coefficient (SMN in beta and gamma frequency bands; *p* = 0.0009) compared with the healthy controls post-treatment. In addition, they demonstrated significantly higher activity in IC-9 coefficient (dual-process of memory perception network, functionally related to a negative correlation between the left VVP and the precuneus in alpha frequency band) after rTMS (*p* = 0.00002).

#### Comparison of Patients vs Controls Post-treatment

After treatment, there was a significant difference observed between participants with bipolar disorder and the control group in SMN and the memory perception network. Patients with BPAD after intervention exhibited significantly higher activity in IC-9 (dual-process of memory perception network, functionally related to a negative correlation between the left VVP and the precuneus in alpha frequency band; *p* = 0.003) and significantly less activity in IC-10 (SMN in beta and gamma frequency bands; *p* = 0.00002).

## Discussion

Our study demonstrated noticeable changes in RSNs activity in patients with bipolar depression following bilateral rTMS to DLPFC. Compared to the control group, responders to rTMS treatment showed significantly lower DMN activity at baseline assessment and non-responders did not significantly differ from the control group at baseline. However, responders showed a significant decrease in SMN connectivity and higher activity in the SMN, visual network, and visual perception network compared to controls was observed in non-responders following treatment. Bilateral rTMS resulted in significant changes in executive functions, verbal memory, and depression symptoms in patients with bipolar depression.

### DMN and SMN Changes at Baseline and Following Bilateral Stimulation Among Responders

In the present study, a reduction of gamma activity in pre-SMA and beta activity in the postcentral area occurred among the responders to rTMS. The results were in line with those in a previous study on patients with bipolar depression, in which gamma frequency activity in postcentral areas was significantly decreased among the responders to unilateral rTMS stimulation (Kazemi et al., 2016). In another study, an imbalance was observed in the DMN/SMN activity of bipolar patients, compared to other resting networks such as DMN/SN and DMN/CEN. Further, a high ratio of DMN/SMN activity was reported in the depression phase while the opposite happened in the manic phase. The relationship between these two networks was considered as a diagnostic marker for this disorder (Martino et al., 2016). The activity of the pre-SMA and precentral areas of SMN is probably related to motor and sleep functions in bipolar patients. Sleep disorders and psychomotor problems are regarded as two predictors of response to rTMS treatment (Brakemeier et al., 2007). Furthermore, the symptom of psychomotor retardation and agitation among depressed patients is a predictor of response to drug therapy (Yoshimura et al., 2004; Mallinckrodt et al., 2007; Herrera-Guzman et al., 2008), ECT (Van Diermen et al., 2015), and rTMS (Brakemeier et al., 2007). Regarding the patients with major depressive disorder (MDD), psychomotor retardation is related to the changes in the integrity of pre-SMA and SMA-proper white matter (pathway), as well as the changes in structural connectivity in rACC-pre-SMA and DLPFC-pre-SMA (Bracht et al., 2012). Some abnormalities were observed in the motor cortex of patients with bipolar disorder when they were doing motor tasks. In addition, an increase in rCBF in the right SMA was reported among these patients (Berns et al., 2002; Caligiuri et al., 2004). The medications used to manage the symptoms of bipolar disorders suppress the activities across motor cortical regions with greater effects in primary motor cortex areas (Caligiuri et al., 2004). Suppressing motor cortex activity among the patients treated by mood-stabilizing medications is regarded as a positive predictor for treatment in bipolar disorder (Caligiuri et al., 2004).

The precentral area is considered as another part of SMN, in which the activities are negatively related to DMN activities (Tomasi and Volkow, 2011). In some studies, sleep disturbance has been considered as a predictor for the response to rTMS treatment (Brakemeier et al., 2007). However, insomnia, as the most common problem, can affect the patients with bipolar depression (Winokur et al., 1969; Casper et al., 1985). Patients with primary insomnia experience some defects in the size of brain gray matter in precentral and postcentral areas (Joo et al., 2013). The activity in the beta frequency band is related to both types of insomnia. Further, an increase in beta activity was observed among the patients with insomnia and healthy people at the onset of sleep and during the NREM phase in sensorimotor areas (Wang et al., 2005). A reduction in the beta activity indicates a decrease in the activities in the postcentral area, and the changes in this area can be related to sleep problems among patients.

### The Changes in Resting State Networks Among Non-responders to Bilateral rTMS

Regarding the non-responders to rTMS, some changes were observed in IC-4, IC-9, and IC-10 after the treatment. These patients experienced a decrease in alpha frequency activity in the areas related to occipital visual network, compared with the healthy controls. In addition, an increase occurred in the IC-9 activities, i.e., increased alpha frequency activities in precuneus and VVP.

A small number of analyses have focused on investigating the electrophysiological correlates of not responding to rTMS treatment (Arns et al., 2012, 2014). These studies have largely focused on EEG power rather than connectivity analysis. For example, non-responders had slower alpha peak at baseline(Arns et al., 2012). Recently, in another study, non-responders to rTMS treatment had low connectivity within the dopaminergic pathway, which is correlated to anhedonia (Downar et al., 2014). However, in the present study, anhedonia was not directly measured. Thus, future studies can focus on anhedonia as a predictor of not responding to treatment in bipolar patients.

### Difference in DMN Functions Between Bipolar Patients and Healthy Controls at Baseline

Less beta and alpha activity at the baseline was observed in mPFC and right TPJ, respectively, among all patients, compared to those in the healthy group. Few studies have evaluated resting EEG abnormalities in bipolar patients. Regarding the results of previous research, the present study supports the role of alpha and beta frequency bands in bipolar disorder (Ozerdem et al. 2008; 2013). Recently, source localization analysis was studied to compare depressive and manic phases in bipolar patients. Based on the results, bipolar patients in the manic phase had lower theta in brodmann areas 13, 38, and 47, compared to bipolar patients in the depressive phase. In addition, higher Beta-2 and Beta-3 were reported in brodmann area 6 and cingulate cortex among the patients. In line with the results in the present study, this study emphasized the role of frontal and temporal lobes in both phases of bipolar disorder (Painold et al., 2014). The results of previous studies indicated that some problems are available in the DMN function (Brady et al., 2017) and the affective network (Luking et al., 2011; Pannekoek et al., 2014) among the patients with mood disorder. According to a recent meta-analysis, there is a hyper-connectivity among depressed patients, compared to healthy people in the socio-affective network (Schilbach et al., 2014). The findings indicate that the abnormalities of these two areas of DMN can be related to the etiology of bipolar depression, which can be regarded as a probable neuromarker.

### Improvements in Cognitive Functions

Considering the literature, the present study pioneered to explore the effectiveness of bilateral stimulation treatment on improving cognitive functions among patients with bipolar depression. However, the findings of this study are consistent with those addressing bilateral stimulation on unipolar patients, which have indicated that rTMS could produce improvements in cognitive functions (Loo et al., 2003; McDonald et al., 2006; Fitzgerald et al., 2012). Further, some significant changes took place in verbal memory, which are consistent with the results of the present study.

Furthermore, the impairment of verbal memory is considered as the only cognitive problem which continues during mania phase, depression, and euthymic mood in patients with BPAD (Basso et al., 2002). An impairment in verbal memory can be considered as a unique feature of bipolar depression, as well as the endophenotype of this disorder, due to its persistence in depressive phase (Malhi et al., 2007). Generally, successful treatment methods have similar effects on the neuropsychological profiles among these patients in treating bipolar depression. Pharmacological treatment (lamotrigine) (Pavuluri et al., 2010) and other therapies (McIntyre et al., 2012) improve the executive functions or verbal memory. Electrophysiological studies demonstrated that cognitive deficits in bipolar patients are related to frontal-temporal dysfunctions (Andersson et al., 2008). In a normal verbal memory, apart from optimal performance in the temporal lobe, the cooperation of frontal lobe, especially ventro mPFC, is required (Gilboa et al., 2009).

The present study demonstrated significant improvement in executive functions after rTMS treatment, in addition to the improvement in verbal memory. Usually, normal function in WCST requires a proper functioning in the prefrontal cortex. The area which is mostly associated with the preservation errors is the DLPFC (Berman et al., 1995). Further, neuroimaging studies have also focused on another area of the frontal cortex called “ventrolateral prefrontal cortex,” which is related to the performance of this test (Konishi et al., 1998). Some studies emphasized that this area of the brain experiences some abnormalities such as a reduction in the volume of gray matter among bipolar patients (Ellison-Wright and Bullmore, 2010). In addition, the stimulation of this area instead of DLPFC has been recently suggested for increasing the response to treatment in bipolar patients (Downar and Daskalakis, 2013).

### Limitations of the Study

One serious limitation of our study was simultaneous use of medicine and rTMS, which made the interpretation of results complex and difficult. But to overcome this problem, patients’ medications remained unchanged from 1 month before treatment to the end of treatment. Furthermore, eLORETA network template was extracted from medicine-free normal patients. Thus, the potential medicine effects on neurophysiological activities could not be completely discounted. To the best of the authors’ knowledge, no previous study has demonstrated medication effects on eLORETA ICA analysis. Nevertheless, cautious interpretation of our results is required. Another potential limitation of this study was the reliance on the BDI-II to evaluate treatment outcomes. However, in previous research where expert-based assessment tools were used alongside self-reports, no significant difference was observed between these methodologies in their evaluation of treatment response and recovery rates (Pridmore et al., 2000). In a previous study, we used 80 healthy subjects with a wide age range (44.2 ± 20.0 years) and revealed that there were common five EEG-RSNs across a wide age range and their activities showed no age dependences (Aoki et al., 2015). This result indicates that age related changes of EEG-RSN activities are better described by cognitive functions rather than age itself which was suggested by fMRI studies (Balsters et al., 2013; Staffaroni et al., 2018). Therefore, in this study, although there was a significant age difference between these 80 healthy subjects and bipolar patients, we could compare EEG-RSN activities between 80 healthy subjects and bipolar patients.

## Conclusion

Targeting the main nodes of SMN and DMN with rTMS appears to be useful in the treatment of depression. The current study suggests targeting the mPFC and TPJ areas of DMN, areas related to the socio-affective network, can be effective in treating bipolar depression.

## Ethics Statement

This study was carried out in accordance with the recommendations of the Declaration of Helsinki, University of Tehran ethics committee. The protocol was approved by the University of Tehran ethics committee.

## Author Contributions

RK and RR designed the study. RK, RR, MR, and SK carried out the study. GB, MH, YA, RI, and MI analyzed the EEG data. RK, MH, RI, and PF interpreted the results and wrote the manuscript. All authors read and approved the final manuscript.

## Conflict of Interest Statement

The authors declare that the research was conducted in the absence of any commercial or financial relationships that could be construed as a potential conflict of interest.
